# The influence of bilingualism on the assessment and treatment of an Italian–English speaker with the logopenic variant of PPA

**DOI:** 10.21203/rs.3.rs-7835928/v1

**Published:** 2025-10-17

**Authors:** Roberta Tomasoni, Gaia C. Santi, Serena Tagliente, Chiara Griseta, Simona Aresta, Allegra Benzini, Paola Santacesaria, Cinzia Palmirotta, Maria Luisa Mandelli, Stephanie Grasso, Petronilla Battista

**Affiliations:** Istituti Clinici Scientifici Maugeri IRCCS Bari; Istituti Clinici Scientifici Maugeri IRCCS Bari; Istituti Clinici Scientifici Maugeri IRCCS Bari; Istituti Clinici Scientifici Maugeri IRCCS Bari; Istituti Clinici Scientifici Maugeri IRCCS Bari; Istituti Clinici Scientifici Maugeri IRCCS Bari; Istituti Clinici Scientifici Maugeri IRCCS Bari; Istituti Clinici Scientifici Maugeri IRCCS Bari; Memory and Aging Center, University of California San Francisco, San Francisco, California, USA; Department of Speech, Language and Hearing Sciences, University of Texas at Austin, Austin, TX, USA; Istituti Clinici Scientifici Maugeri IRCCS Bari

**Keywords:** Primary progressive aphasia, bilingual experience, multilingualism, cross-linguistic transfer, lexical retrieval therapy, bilingual speech-language assessment, bilingual speech-language intervention

## Abstract

**INTRODUCTION:**

The impact of bilingualism on speech-language assessment and therapy in Primary Progressive Aphasia (PPA) remains underexplored, despite its suggested influence on disease presentation.

**METHODS:**

A bilingual Italian (L1)-English (L2) individual with logopenic-variant PPA completed a bilingual assessment and dual-language Lexical Retrieval Therapy. His bilingual experience was characterized, and therapy outcomes were evaluated within and across languages.

**RESULTS:**

Pre-morbidly, the patient was a relatively balanced bilingual with stronger L1 literacy. Following disease onset, L2 showed faster decline, while L1 remained stable. Age of acquisition, dominance, and language use were among the main factors contributing to language maintenance and decline. Therapy yielded equivalent item-specific gains in both languages (L1 d_2_ =5.0; L2 d_2_ =5.6), asymmetric cross-linguistic transfer to L2 > L1, and modest functional improvements.

**DISCUSSION:**

Findings support the relevance of in-depth bilingual assessment and therapy underpinning successful treatment in PPA, unveiling the significance of bilingual experience in shaping treatment outcomes.

## 1. INTRODUCTION

Bilingualism can reshape neurodegenerative trajectories by bolstering cognitive reserve, enhancing executive control and attentional flexibility and has been linked to delayed dementia onset [1]. Its effects in language-led dementias (PPA) are understudied: retrospective data suggest a ~ 4-year delay in onset of logopenic variant of PPA (lvPPA), with smaller, variable delays in non-fluent (nfvPPA) and semantic PPA (svPPA) [2]. Existing work in bilingual PPA shows parallel or asymmetric L1/L2 decline, with L1 consistently better preserved than L2, shaped by age of acquisition, premorbid and postmorbid proficiency. However, the lack of systematic language-sensitive assessments in both languages is detrimental to capturing patients’ cognitive-linguistic strengths and impairments, thereby limiting a comprehensive tracking of the evolution of individual abilities. In the same vein, few studies have examined how bilingualism impacts outcomes in speech and language therapy (SLT) for individuals with this syndrome [3]. The effectiveness of SLT can depend on the individual’s proficiency in each language, with therapy potentially being more successful in the more dominant language. Evidence suggests that bilingual patients may experience comparable or even better outcomes when therapy is appropriately adapted, as skills acquired in one language can sometimes transfer to the other, facilitating recovery or maintenance of language functions across both languages [4–5]. In this case report, we characterized the bilingual profile of an Italian-English lvPPA participant by developing an in-depth functionally equivalent bilingual assessment and a tailored Lexical Retrieval Therapy (LRT) administered in Italian and English. The relationship between bilingual experience with speech and language profile before and after treatment was explored by considering the most relevant bilingual features in shaping L1 and L2 performance. We analyzed whether intervention leads to within- and cross-linguistic improvement in (i) naming; (ii) formal speech and language assessment; (iii) connected-speech production.

Based on prior work, we hypothesised that (1) baseline performance would be modulated by age of acquisition, with more preserved L1 abilities [6]; (2) LRT would yield gains in both L1 and L2, with greater cross-linguistic transfer (CLT) to L1 [7–9] in the context of more stable performance on language assessments for L1 compared to L2 after therapy; and (3) generalisation effects would extend beyond lexico-semantic tasks to functional communication although evidence in PPA is inconsistent [10, 11].

## 2. METHODS

### 2.1 Case history

**Figure 1.**
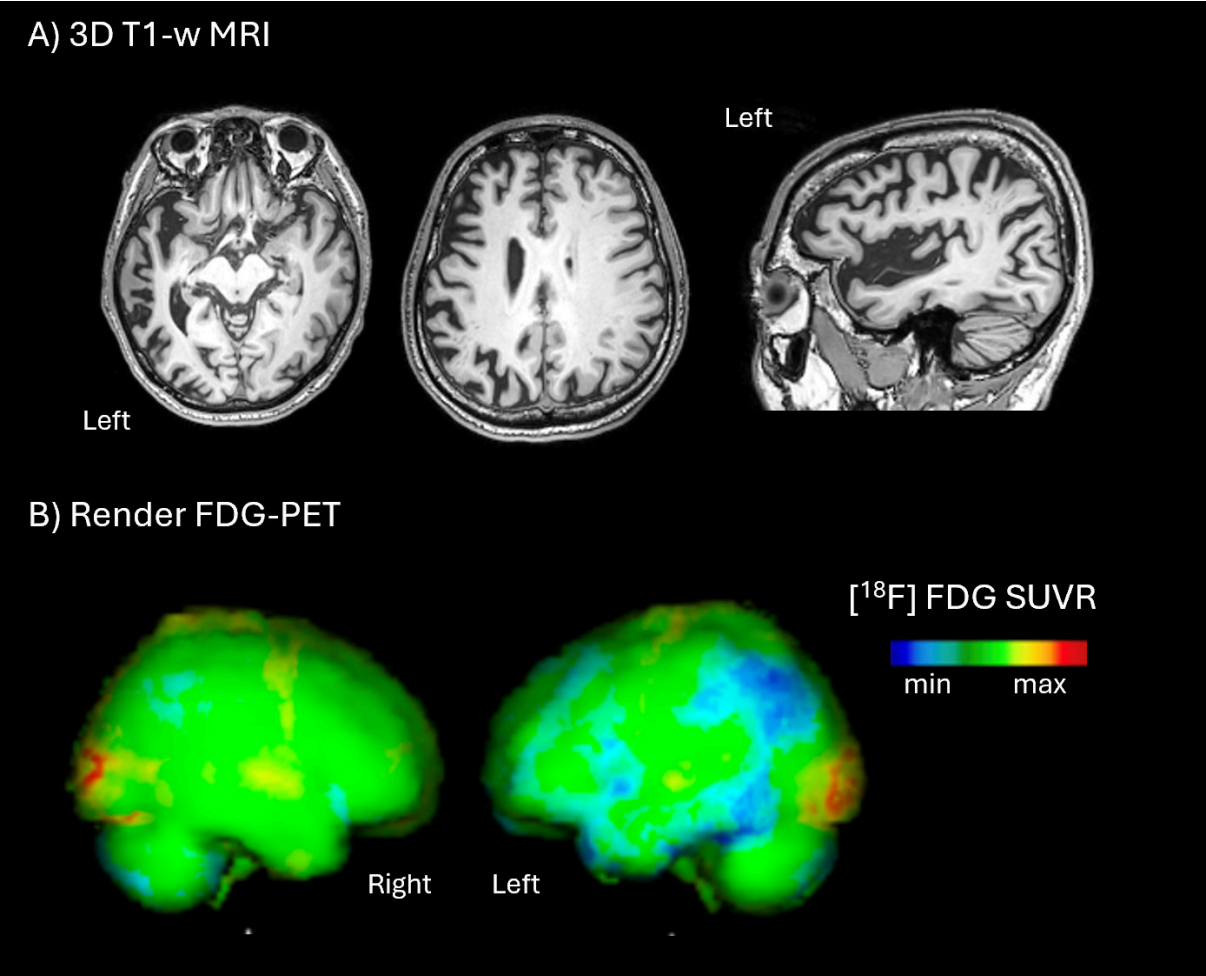
Imaging. **(A)** T1-weighted MRI demonstrates predominantly left-sided atrophy in frontal and temporo-parietal regions. **(B)** PET FDG shows hypometabolism (blue areas) in the left posterior temporo-parietal and inferior frontal cortices.

In April 2025, a bilingual speaker of Italian and English (L.R.), a right-handed 60-year-old male with eighteen years of formal education, was referred to our Laboratory of Neuropsychology by his family after a 2-year history of progressive language deficits. He was diagnosed with lvPPA in 2024 according to the criteria of Gorno-Tempini et al. (2011) [12]. Genetic testing identified a heterozygous 6-bp frameshift deletion in exon 6 of the GRN gene (NM_002087.4). A positive family history of early-onset neurodegeneration was reported. Baseline neuropsychological assessment in Italian showed mild impairments in verbal episodic memory and visuospatial skills, with a moderate executive deficit. On the Frontal Behavioural Inventory (FBI) [13], moderate disorganization and inattention were noted, while activities of daily living remained intact. MRI (3T) demonstrated left-greater-than-right temporo-parietal atrophy (Fig. 1A). FDG-PET showed hypometabolism in the left posterior temporo-parietal and inferior frontal cortices (Fig. 1B).

### 2.2 Speech and language assessment

To fully characterise L.R.’s speech and language profile, a structured assessment protocol was administered in Italian and English before and after LRT (Table S1 of Supplementary Material). The protocol assessed articulatory, phonetic–phonological, lexico-semantic, morphosyntactic and discourse domains. When parallel validated tools were not available across both languages, functionally equivalent assessments were adopted (e.g., Screening for Aphasia in Neurodegeneration, SAND [14, 15] was compared to the equivalent Mini-Linguistic State Examination, MLSE [16]). Since one of the patient’s main language difficulties related to naming, developing an ad hoc naming task was essential to directly compare lexical retrieval in the two languages. Thus, an experimental 20-item task was developed in Italian and English. Items consisted of 10 living and 10 non-living nouns, balanced across frequency tiers (low, medium, high) and matched for word length and semantic category. Italian words were selected from the COLFIS database [17] and English words from Celex [18]. Frequency groups were defined using log10 of frequency, and translation equivalents were carefully matched across languages. All images were selected from the BOSS database [19] and the same images were probed in Italian and English. Stimulus lists and lexical characteristics are provided in Supplementary Material (Table S2). Connected speech was elicited with the “Cat Rescue” picture [20], and self-perceived communication outcomes were assessed with the Communication Outcomes After Stroke (COAST) questionnaire [21].

### 2.3 Bilingual Language Background

Bilingual experience was assessed with the Bilingual Language Profile (BLP) [22] and the Language History Questionnaire (LHQ-3) [23]. The BLP provided a quantitative index of pre and post-morbid dominance, proficiency, and usage, while the LHQ-3 detailed age of acquisition, frequency of use, self-rated proficiency, and sociolinguistic context. These complementary instruments were selected to provide both quantitative pre and post-morbid dominance indices (BLP) and qualitative developmental context (LHQ-3) necessary for comprehensive bilingual profiling in progressive aphasia.

### 2.4 Treatment design

A single-subject, multiple baseline experimental design was adopted to assess the effect of language intervention on L.R., with a focus on within-language gains and potential CLT.

The treatment lasted five weeks, with three one-hour sessions per week and daily home practice. Therapy was delivered in three stages: two weeks in Italian (L1), two weeks in English (L2), and a final review week in both languages to mitigate potential effects of temporal delay between the initial Italian baseline assessment and the subsequent post-therapy evaluations. Sessions were provided remotely using Microsoft Teams by a bilingual speech-language therapist qualified in both the UK and Italy (RT). Therapy was delivered in L1 first to facilitate comprehension of the therapy task.

**Figure 2.**
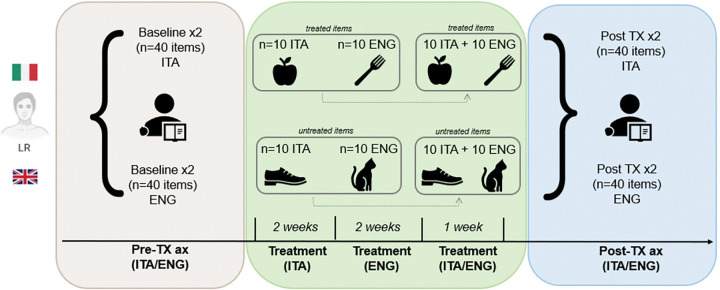
Study Design Flowchart. Two baseline assessments (n = 40 items) were collected pre-therapy in both Italian and English. The treatment phase included Phase 1 (Italian, 2 weeks, 20 items, 10 treated and 10 untreated) and Phase 2 (English, 2 weeks, 20 items, 10 treated and 10 untreated), followed by a 1-week review of all items in both languages. In the post-therapy phase, all items were probed twice in both languages.

The study design flowchart is presented in Fig. 2.

Therapy followed an adapted LRT following the Lexical Retrieval Cascade Treatment protocol [24, 25] including eight steps: (i) naming; (ii) semantic self-cue using semantic prompts; (iii) orthographic cue (first syllable); (iv) reading; (v) writing and repeating; (vi) semantic plausibility judgment; (vii) word recall; (viii) generalisation tasks (i.e., sentence production using the item in context). Daily homework used a copy-and-recall procedure [26].

In order to select the therapy items, 155 functional items (selected by the participant and his partner) were probed twice in each language at baseline for naming accuracy. From this pool, 40 items were selected to form four sets of five words in each language: two trained and two untreated. Selection prioritized items named incorrectly or inconsistently at baseline and were balanced for word frequency, length, syllable count, and semantic category (e.g., food, hygiene, clothing). Each therapy set was treated for one week, so items were treated during three sessions. In each therapy session, half of all items - five treated and untreated - were elicited to ensure regular exposure.

### 2.5 Statistical analysis

The bilingual experience was derived from the scoring of LHQ3 and BLP questionnaires. To fully characterize the bilingual profile, we also compared baseline speech and language performance at formal testing in Italian and English using a non-parametric unpaired permutation test, with 10,000 Monte Carlo permutations.

To evaluate whether treatment was effective within and across the two languages, we evaluated the number of correctly named treated and untreated items post-therapy relative to baseline in both languages. Effects were quantified using Percentage of Non-Overlapping Data (PND) and d_2_ effect sizes due to limited data variability [27–30]. Conventionally, PND > 90 is highly effective, < 50 is ineffective; d_2_ values of 2.6, 3.9, and 5.8 indicate small, medium, and large effects, respectively. CLT was defined as any improvement obtained at the naming task for treated items in the untreated language (e.g., training “burro di arachidi” in Italian, leading to the correct naming of “peanut butter” in English). We also investigated pre–post changes in Italian and English across formal speech and language testing, performing a paired permutation test (10,000 iterations).

To assess generalization effects (i.e., the patient’s lexical retrieval ability in non-naming tasks), two independent raters extracted and qualitatively compared connected-speech measures of lexical density (ratio of nouns/verbs to total words) and syntactic complexity (subordinate clauses per utterance) [31, 32] pre and post-treatment.

Finally, COAST scores were directly compared before and after therapy to assess self-perceived changes in functional communication.

## 3. RESULTS

### 3.1 Bilingual language background

L.R. grew up in an Italian–Japanese family, exposed to Italian (L1) from birth and English (L2) from age 4. Italian predominated in family and informal contexts, while English was primarily used in school and work. In adulthood, he acquired oral fluency in French and passive knowledge of Japanese without literacy. At the time that this study was conducted, L.R. did not report any use of French and Japanese. Pre-morbidly, Italian and English oral proficiency was relatively balanced, with stronger literacy in Italian. After symptom onset, English (L2) use declined, with Italian remaining the dominant and less impaired language (Table 1).

### 3.2 Speech and language characterization

At baseline, L.R. exhibited deficits in line with lvPPA. Naming and sentence repetition were predominantly impaired, with English (L2) naming (p = 0.038) and reading (p < 0.001) performances worse than Italian (L1). The patient also showed simplified sentence structure and argument-structure errors, especially in L2 (p = 0.044). Connected speech showed reduced lexical density in L2 compared to L1, with contextual comprehension relatively preserved across languages.

### 3.3 Treatment

**Figure 3.**
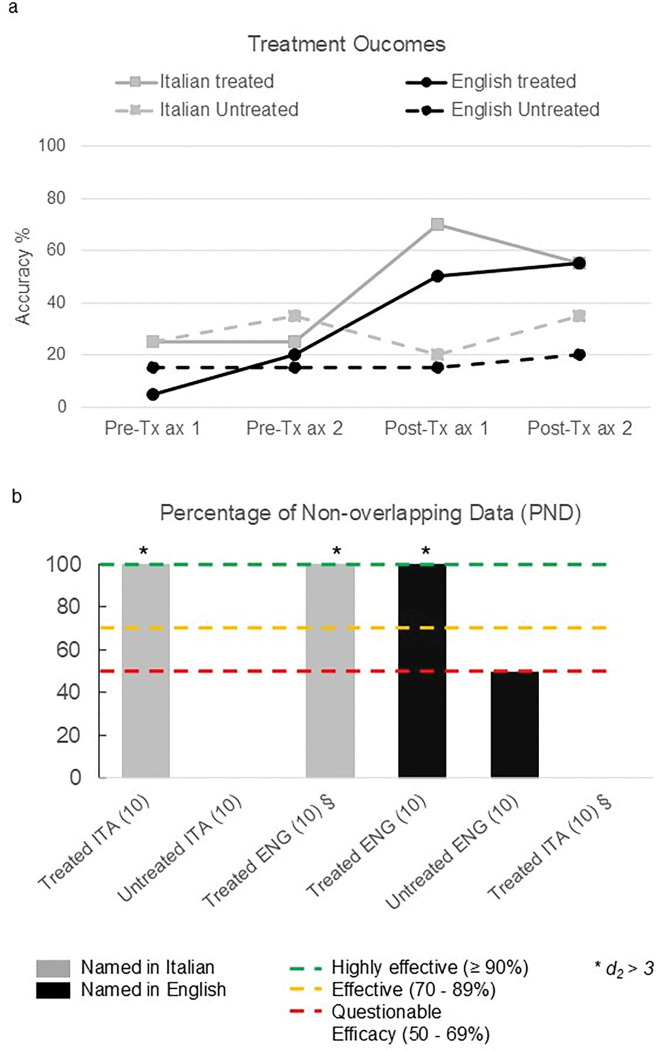
Summary of results on treated and untreated items in Italian and English. The figure shows the percentage of non-overlapping data (i.e., the number of times that the patient performed better at post-treatment probing compared to pre-treatment probing). Grey columns refer to the two baseline assessments performed in Italian, and black columns refer to the ones in English.

Therapy produced item-specific gains in both Italian and English. PND values indicated medium-to-large effect sizes (Italian d_2_ = 5.0; English d_2_ = 5.6) (see Figure 3a). Untreated items showed no improvement (PND = 0–50). CLT was asymmetric: items treated in English improved when probed in Italian (PND = 100; d_2_ = 3.0), but no transfer occurred from Italian to English (PND = 0) (See Figure 3b). Overall performance was maintained post-treatment. Within-language comparisons showed a trend toward improvement in Italian NAT (p = 0.063) and significant gains in English argument structure production (NAVS; p = 0.008). Despite these improvements, English syntactic abilities showed an overall tendency toward decline (Table 2). Between-language comparisons revealed significantly lower scores in English than Italian for reading (p = 0.005) and morphosyntactic tasks, including NAVS sentence production priming, NAT, and syntax comprehension (all p < 0.05) (Table 3). Qualitatively, at baseline, connected speech was more preserved in Italian than in English for lexical density (35.17 and 14.18, respectively). After therapy, English showed broader improvements, with lexical density nearly doubling (from 14.18 to 30.21) and verb production increasing in both languages (from 32 to 37 in Italian, from 21 to 48 in English). Syntactic complexity diverged: it increased in Italian (greater use of subordinate clauses, scoring from 0.69 to 1) but decreased in English (from 1.36 to 0.64). Finally, COAST scores improved from 62.5% to 73.3%, indicating a modest but subjectively meaningful functional benefit in the following areas: speaking with a familiar interlocutor, a reduction of the impact of language difficulties on family life, social life, and hobbies.

## 4. DISCUSSION

This single-case study shows that an extensive assessment of bilingual experience was crucial since it allowed identifying age of acquisition, dominance, and language use as main factors contributing to language maintenance and decline in our bilingual patient with lvPPA. Moreover, we found that a structured LRT can improve naming of treated items in both languages. Notably, greater gains were observed in L1 because of the cross-linguistic transfer resulting from treatment of L2. The naming improvement came in the context of a stable speech-language profile in both languages. With respect to generalization to other linguistic tasks (i.e., connected speech), L.R. ‘s lexical density improved, with a greater number of nouns produced in L2 and of verbs retrieved in both languages. In functional communication, measured by the COAST scale, L.R. reported a small but personally significant improvement in interaction with familiar speakers and in social participation.

Before treatment, L.R. was more impaired in English (L2) than Italian (L1), which echoes previous reports of faster decline in the less dominant, second-learned language [6]. This pattern cannot be explained by age of acquisition alone: his pre-morbid dominance, patterns of use, and stronger literacy in Italian appear to have offered greater protection for L1. The drop in his confidence and use of English after symptom onset may have further accelerated L2 decline. These observations align with the Adaptive Control Hypothesis [33], which posits that bilinguals recruit domain-general cognitive control networks, including prefrontal, anterior cingulate, and basal ganglia circuits, to manage processes of language selection, inhibition, and switching. Neurodegeneration may differentially affect these networks, reducing the efficiency of control and thereby increasing the likelihood that the less practiced or less dominant language becomes more vulnerable to decline [34]. In L.R. ‘s case, this may account for both the steeper post-morbid decline in English and the asymmetric transfer, with the weaker language driving maintenance in the stronger one.

LRT led to substantial item-specific gains in both languages and CLT from English to Italian. The direction of transfer is noteworthy. The Revised Hierarchical Model [35], often used to explain CLT in bilingual speakers, predicted stronger influences of L1 on L2. However, studies on bilingual healthy populations revealed that lexical retrieval tasks activate the same semantic networks across languages. When bilinguals named items, they presented with a similar degree of semantic interference in L1 and L2, suggesting that semantic control abilities are independent of the language being utilised [36]. Accordingly, evidence from post-stroke aphasia [37] and PPA literature [4] also highlighted greater CLT from L2 to L1, suggesting that shared semantic representations can facilitate improvement even when the treated language is comparatively less dominant. This is in line with dual-language intervention studies evidencing that targeting the weaker language seems to benefit both L1 and L2, supporting broader communication gains [4, 38]. Our results align with the latter findings, suggesting a benefit in training L2 and L1, leading to greater gains in L1 by activating the same shared semantic network. This is further confirmed by a recent meta-analysis by Lee & Faroqi-Shah (2024) [38], which reported medium-to-large effect sizes for dual-language anomia treatment in aphasia. Our d_2_ values (≈ 5.0–5.6) fall squarely in that range, which adds weight to these findings despite the single-case design.

Additionally, existing literature suggests that the asymmetry of transfer may be influenced by other factors, including which language is treated and in what order, the relative pre- and post-morbid dominance and proficiency of the two languages, and how frequently and confidently they are used [9, 39, 40]. In PPA, differential patterns of language decline can shape confidence and frequency of use in the more impaired language, which may in turn influence cross-linguistic treatment effects [4]. Accordingly, in the present case, L.R. ‘s dominance and frequency of use for L2 presented a slight imbalance compared to L1 pre-morbidly and a significant reduction of frequency, dominance, confidence and use post-morbidly. This might have contributed to the lack of transfer from L1 to L2.

Changes in connected speech mirrored the main therapy results: lexical density qualitatively improved in both languages, with particularly striking gains in English. Syntax followed a different trajectory, improving in Italian but declining in English, which likely reflects the combined effects of disease progression and the item-specific focus of therapy. Improvements on argument-structure tasks support this interpretation, as they were indirectly targeted during generalization activities. Healthy controls typically produce picture descriptions that are considerably more content-rich and syntactically more complex than those of individuals with aphasia [41, 42]. Against this backdrop, L.R.’s English lexical density improved from ~ 14% at baseline to ~ 30% post-therapy, approaching the level typically seen in healthy discourse. Although direct comparisons for Italian should be interpreted with caution, given the lack of Italian-specific normative data for the “Cat Rescue” connected speech task. Beyond behaviour, there may also be a neurobiological explanation. Luk et al. (2011) [43] showed that lifelong bilingualism helps maintain white-matter integrity in older adults. This preservation may underlie the resilience we observed in L.R.’s connected speech, particularly when coupled with the modest but meaningful boost in self-reported communication. Taken together, these results suggest that bilingual experience might support language performance both through behavioral strategies and through underlying neuroprotective mechanisms. In addition, these results provide candidate measures derived from connected speech that may prove useful in detecting treatment response in the study of PPA treatment more broadly.

Several limitations must be acknowledged. The single-case design, small treatment set, and binary scoring limited our analyses’ variability and statistical power. In addition, not all assessments were fully matched across languages. Although this was a pragmatic choice given the lack of equivalent Italian–English tools, international guidelines on bilingual practice [44] stress the importance of matched assessments and cultural competence, pointing to an area for future improvement in the field more generally.

It is also worth situating this report within the broader field. A recent scoping review of bilingual PPA [3] emphasised the limited systematic evidence on therapy outcomes in this population. This is further compounded by several barriers in the Italian context, where accessing SLT services is challenged by long waiting times, reduced awareness, and limited experience of the SLT role in managing people with PPA, and insufficient funding in providing proactive and long-term services [45]. By providing detailed assessment and intervention data, our study contributes to filling this gap. It underscores the need for larger, multi-site studies that can explore therapy responsiveness across different bilingual profiles. Incorporating literacy and neuroimaging measures that correlate with therapy’s effects will also be necessary.

To conclude, this case study illustrates how bilingual experience shapes not only the trajectory of language decline in PPA but also the effectiveness of therapy. In L.R., Italian was preserved longer, likely supported by greater dominance, literacy and use, while English declined steeply yet provided the entry point for cross-language transfer. These findings extend earlier work showing that lifelong bilingualism contributes to cognitive reserve and shapes cognitive profile in the case of neurodegenerative onset [46, 47]. They are also consistent with the Adaptive Control Hypothesis [33], which frames neurodegeneration as a disruption of language-control networks. Integrating these perspectives makes it clear that bilingual profiles must be considered when planning therapy. For aging bilingual populations, cross-linguistic treatment approaches and bilingual service provision are not optional but essential to ensure equitable and effective care.

## Supplementary Material

Supplementary Files

This is a list of supplementary files associated with this preprint. Click to download.

• SupplementaryMaterials1.docx

## Figures and Tables

**Table 1.** Bilingual language background.

LHQ3	Italian	English	French	Japanese	
Spoken language Learning	0.975	0.966	0.941	0.27	
Written Language Learning	0.962	0.958	0.937	0.266	
Spoken Language Proficiency	0.928	0.857	0.714	0.258	
Written Language Proficiency	0.714	0.714	0.642	0.285	
Spoken Language Usage	0.906	0.5	0.281	0.281	
Written Language Usage		0.187	0.062	0.062	
Spoken Language Dominance	0.583	0.446	0.338	0.177	
Written Language Dominance	0.466	0.45	0.352	0.174	
Spoken Language Dominance Ratio	1	0.764	0.579	0.305	
Written Language Dominance Ratio	1	0.966	0.755	0.73	
BLP	Italian	English	French	Japanese	
Linguistic History	95	77	35	5	
Language use before symptoms	30	18	2	0	
Language use currently	40	10	0	0	
Linguistic competence before symptoms	24	21	13	1	
Linguistic competence currently	18	14	10	0	
Language attitude	20	17	8	2	
Global score before symptoms	175.1	140.8	65.7	9	
Global score currently	172.99	116.2	56.7	6.8	
	Ita-Eng	Ita-Fre	Eng-Fre	Ita-Jap	Eng-Jap
Dominance Before Symptoms	34.8	109.9	75	166.6	131.7
Dominance Currently	56.7	116.2	59.4	166.1	109.4

Linguistic profile from the LHQ3: Language History Questionnaire (unpublished in Italian) and the BLP: Bilingual Language Profile.

**Table 2.** Within-language comparisons of pre- and post-treatment assessment scores.

Speech and	Language Test	Italian T0 (pre)	Italian T1 (post)	p-value	English T0 (pre)	English T1 (post)	p-value
**SAND**	**MLSE**				-	-	
-	*Articulation*	-	-	-	1/3	3/3	0.500
*Naming*	*Naming*	9.5/14	7/14	0.625	2/6	3/6	1
*Single word*	*Single word*						
*comprehension*	*comprehension*	12/12	10/12	0.500	2/3	2/3	1
*Sentence*	*Sentence*						
*comprehension*	*comprehension I*	8/8	7/8	1	3/4	2/4	1
	*Sentence*						
-	*comprehension II*	-	-	-	3/4	2/4	1
*Single word*	*Single word*						
*repetition*	*repetition*	10/10	9/10	1	5/6	4/6	1
*Sentence repetition*	*Sentence repetition*	2/6	3/6	1	1/4	1/4	1
*Reading*	*Reading*	15/16	15/16	1	1/10	4/10	0.250
*Writing*	*Writing*	4/6	4/6	1	N.A	N.A	
*Semantic*	*Semantic*						
*association*	*association*	3/4	3/4	1	4/4	4/4	1
*Picture description*	*Picture description*	8/8	7/8	1			
**NAVS ITA**	**NAVS ENG**						

*Verb naming*	*Verb naming*	18/22	14/22	0.289	14/22	15/22	1
*Verb comprehension*	*Verb comprehension*	22/22	22/22	1	22/22	22/22	1
*Argument structures production*	*Argument structures production*	30/32	30/32	1	23/32	31/32	***0.008***
*Sentence production priming*	*Sentence production priming*	21/30	25/30	0.388	17/30	14/30	0.506
*Sentence comprehension test*	*Sentence comprehension test*	24/30	25/30	1	25/30	24/30	1
**NAT ITA**	**NAT ENG**	16/22	21/22	0.063	18/30	15/30	1
**Ad hoc picture naming ITA**	**Ad hoc picture naming ENG**	14/20	11/20	0.250	11/20	12/20	1
**Pyramids and Palm Trees ITA**	**Pyramids and Palm Trees ENG**	14/14	12/14	0.500	10/14	9/14	1
**Syntax Comprehension ITA**	**Syntax Comprehension ENG**	42/48	46/48	0.219	40/48	38/48	0.774
**ENPA**					-	-	
*Words repetition*		14/15	15/15	1			
*Sentences repetition*		3/3	3/3	1			
*Numbers repetition*		10/10	9/10	1			

**WAB**										
*Sentences repetition*				0.625		86/100		80/100		0.625
**CAGI**							-		-	
*Naming*		23/48	22/48		1					
*Comprehension*		46/48	48/48		0.500					
**SAT**		72/76	73/76		1					
**Picture Description ITA**	**Picture Description ENG**									
*Total words*	*Total words*	188	176				111		220	
*Total nouns*	*Total nouns*	35	31				14		30	
*Total verbs*	*Total verbs*	32	37				21		48	
*Lexical Density*	*Lexical Density*	35.17	31.21				14.18		30.21	
*Syntactic Complexity*	*Syntactic Complexity*	0.69	1				1.36		0.64	

Differences within language were derived from paired permutation tests (10,000 Monte Carlo resamples). N.A: non available data; in grey: permutation not performed.

**Table 3.** Between language comparisons pre- and post-LRT.

Test	n. Item PRE TREATMENT						POST TREATMENT			
ENG ITA Mean Mean ENG-p value							Mean Mean ENG-p value ENG ITA ITA			
ENG ITA ITA										

**SAND MLSE**										

*Naming*	6	14	0.333	0.857	−0.524	**0.038**	0.500	0.714	−0.214	0.610

*Single word*	3	12	0.667	1.000	−0.333	0.203	0.667	0.833	−0.167	1.000

*comprehension*										

*Sentence*	4	8	0.750	1.000	−0.250	0.337	0.500	0.875	−0.375	0.504

*comprehension*										

*Single word*	6	10	0.833	1.000	−0.167	0.381	0.667	0.900	−0.233	0.520

*repetition*										

*Sentence*	4	6	0.250	0.333	−0.083	1.000	0.250	0.500	−0.250	0.577

*repetition*										

*Reading*	10	16	0.100	0.938	−0.838	**0.000**	0.400	0.938	−0.538	**0.005**

*Semantic*	4	4	1.000	0.750	0.250	1.000	1.000	0.750	0.250	1.000

*Association*										

**Ad hoc naming task**	20	20	0.550	0.700	−0.150	0.519	0.600	0.550	0.050	1.000

**Pyramids and Palm**	14	14	0.714	1.000	−0.286	0.099	0.643	0.857	−0.214	0.390

**NAT**	30	22	0.600	0.727	−0.127	0.393	0.567	0.955	−0.388	**0.002**

**NAVS**	22	22	0.636	0.818	−0.182	0.311	0.682	0.636	0.046	1.000
*Verb naming test*										
*Verb*	22	22	1.000	1.000	0.000	1.000	1.000	1.000	0.000	1.000

*comprehension*										

*test*										

*Argument*	32	32	0.719	0.938	−0.219	**0.044**	0.969	0.938	0.031	1.000

*structure*										

*production*										

*Sentence*	30	30	0.567	0.700	−0.133	0.430	0.467	0.833	−0.367	**0.006**

*production*										

*priming*										

*Sentence*	30	30	0.833	0.800	0.033	1.000	0.800	0.833	−0.033	1.000

*comprehension*										

*test*										

*Syntax Comprehension*	48	48	0.833	0.875	−0.042	0.770	0.792	0.958	−0.167	**0.028**

Permutation tests’ results. Mean: proportion of correct answers. ENG-ITA: differences between English and Italian performances, Note: LRT: Lexical Retrieval Therapy; SAND: Screening for Aphasia in Neurodegeneration; NAT: Northwestern Anagram Test
